# Adjunctive Local Application of Lidocaine during Scleral Buckling under General Anesthesia

**Published:** 2011-07

**Authors:** Alireza Dehghani, Kamran Montazeri, Amin Masjedi, Najmeh Karbasi, Leila Ashrafi, Behrooz Saeedian

**Affiliations:** Isfahan University of Medical Sciences, Isfahan, Iran

**Keywords:** Retinal Detachment, Scleral Buckling, Lidocaine, Oculocardiac Reflex

## Abstract

**Purpose:**

To evaluate the effect of local lidocaine application on the incidence of the oculocardiac reflex (OCR) during scleral buckling (SB) for rhegmatogenous retinal detachment (RRD) under general anesthesia.

**Methods:**

In a randomized clinical trial, eyes with RRD scheduled for SB under general anesthesia were randomized to adjunctive local application of 1 ml lidocaine 2% versus normal saline to the muscles after conjunctival opening. Surgical stimulation was initiated 5 minutes afterwards. Additionally, 100 mg of lidocaine 2% was added to 50 ml of normal saline in the treatment group which was used for irrigation during surgery; control eyes were irrigated with normal saline. The incidence of the OCR, rate of postoperative nausea/vomiting (PONV), total intravenous (IV) analgesic dose, duration of surgery, and period of hospitalization were compared between the study groups.

**Results:**

Thirty eyes of 30 patients including 22 (73.3%) male and 8 (26.7%) subjects with mean age of 49.4±16.3 years were operated. OCR and PONV occurred less frequently, and total intravenous analgesic dose was significantly lower in the lidocaine group (P < 0.05 for all comparisons). However, no significant difference was noted between the study groups in terms of duration of surgery and period of hospitalization.

**Conclusion:**

Adjunctive local application of lidocaine during SB under GA for RRD decreases the rate of OCR and PONV, reduces the intravenous analgesic dose, but does not affect the duration of surgery or hospitalization.

## INTRODUCTION

Scleral buckling (SB) which is performed for repair of rhegmatogenous retinal detachments (RRDs) is often associated with postoperative nausea/vomiting (PONV) and severe pain.^1^ The oculocardiac reflex (OCR) during these procedures may cause severe bradyarrhythmias, nodal rhythms, ectopic beats, ventricular fibrillation or asystole,^2^ probably due to traction on extraocular muscles^3^ or pressure on the globe^4^. The OCR is decreased after repetitive stimulations due to fatigue of the reflex arc at the level of the cardioinhibitory center. Repeated interruptions may be required during surgery to abort the reflex; this may prolong the operation and lead to anxiety in the surgical team. Treatment with atropine, although effective in blunting the reflex, may itself result in a variety of cardiac arrhythmias.^5^

Preemptive analgesia, an antinociceptive treatment that prevents activation of the reflex^3^, may decrease postoperative pain and prevent excitability in spinal dorsal horn neurons.^6^,^7^ The timing and efficacy of such treatment, along with the issue of postoperative inflammation, however are still matters of debate.^8^–^10^ Preoperative injections of local anesthetics via retrobulbar,^11^,^12^ peribulbar,^13^–^15^ or subtenon^3^,^16^ routes in patients undergoing vitreoretinal surgery under general anesthesia (GA) have been reported to reduce postoperative pain. They also improve intraoperative hemodynamics as observed in patients receiving preoperative peribulbar^15^ or subtenon^3^,^16^ anesthesia; however, regional blocks are associated with complications such as globe perforation and retinal hemorrhage^17^–^19^, cranial nerve palsies^20^, seizures^21^, cardiorespiratory arrest^22^, restrictive strabismus^23^, retinal vein and artery occlusion^18^,^24^, and injury to the optic nerve.^25^

Possible advantages of intraoperative local application of anesthetics are fast visual recovery, easier and more cost-effective administration, and avoidance of needle-related complications. Topical anesthesia has been widely used for phacoemulsification cataract surgery^26^–^28^ and has proven efficacy for trabeculectomy^29^,^30^, as well as for selected cases of pterygium surgery^31^, corneal laceration repair^32^,^33^, and penetrating keratoplasty^34^,^35^.

Topical lidocaine drops have also been used intraoperatively to decrease the incidence and severity of the OCR^2^,^36^ and to prevent pain and PONV after strabismus surgery^37^ and vitrectomy without scleral buckling^38^. To the best of our knowledge, there is no report on the effectiveness of local anesthetics in SB procedures. The current study was designed to evaluate the efficacy of local lidocaine combined with GA on intraoperative hemodynamics and postoperative pain, and nausea/vomiting in patients undergoing scleral buckling surgery.

## METHODS

This double-blind, randomized clinical trial was conducted at Feiz University Hospital, Isfahan, Iran from 2009 to 2010. The research protocol was approved by the Isfahan University of Medical Sciences Ethics Committee in accordance with the tenets of the Helsinki Declaration and the study was registered at www.clinicaltrials.gov as NCT01417572. Informed consent was obtained from all patients. Study participants had been referred for elective scleral buckling surgery under GA. Inclusion criteria were age > 15 years and class 1 to 3 American Society of Anesthesia (ASA) criteria. Patients with major psychiatric conditions, neurological impairment, history of allergy to the study drugs, previous vitreoretinal or strabismus surgery, intraocular or extraocular inflammation or pain syndromes necessitating treatment with opioids or nonsteroidal anti-inflammatory drugs were excluded. Furthermore, patients with preoperative heart rate (HR) less than 70 or greater than 75 beats per minute (bpm), systolic blood pressure (BP) less than 12 mmHg or higher than 13 mmHg or any sign of heart block on the preoperative electrocardiogram were excluded. Patients who developed intraoperative complications such as subretinal hemorrhage and vitreous hemorrhage were also excluded.

Simple non-random consecutive sampling was employed for case finding. After inclusion, participants were randomized to the treatment or control groups using a computer generated table. Considering alpha error of 0.05, study power of 80, P_1_ = 86% and P_2_ = 20%, fifteen patients were required for each study group.

In the theater reception area, two drops of lidocaine 2% or normal saline were instilled in the eyes of subjects in the treatment and control groups, respectively. Surgery was performed under general anesthesia by the same anesthesiology team, using an identical method by a single surgeon in both groups. After preoxygenation (10 liter flow during 2 minutes with SPO_2_ > 95%), induction was accomplished by intravenous fentanyl 2 microgram/kg, thiopental 5 mg/kg, and atracurium 0.5 mg/kg. Anesthesia was maintained with O_2_/N_2_O (50/50) and isoflurane with inspiratory concentration of 0.5 to 1%. Monitoring was performed by electrocardiography, arterial BP measurement and pulse oximetry.

After opening the conjunctiva, the treatment group received 1 cc of lidocaine 2% applied locally to the muscle and the placebo group received the same volume of normal saline (0.9%). Surgical stimulation was initiated 5 minutes after completion of the previous step. Additionally, 100 mg lidocaine was added to 50ml of saline solution which was used for intraoperative irrigation in the treatment group. The same volume of normal saline was used for irrigation in the control group. Duration of surgery was greater than 45 minutes in all patients; 1/3 of the irrigation fluid was used in each 15 minute period during the first 45 minutes of the procedure.

Scleral buckling started with 360° peritomy. The subtenon space was entered and the rectus muscles were engaged with muscle hooks, followed by insertion of traction sutures. Cryopexy was then applied as necessary. We used segmental or encircling solid silicone rubber (#240 or #287; Mira, Waltham, MA, USA) or silicone sponge (#505; Mira, Waltham, MA, USA) explants. Adjustment of buckle height was achieved by tightening the encircling element or by changing the distance between the scleral sutures.

BP and HR were recorded before and after stimulation of the OCR which was defined as rapid reduction in HR by 20% from baseline or development of arrhythmia during ocular manipulation. Severe OCR was considered as reduction of HR to less than 40 bpm or systolic BP to less than 80 mmHg. OCR was managed by withholding manipulations and intravenous atropine (10 μg/kg) injection if OCR occurred more than three times or in case of a severe reflex.

Study variables included duration of anesthesia and surgery, rates of intraoperative OCR and severe OCR, incidence of postoperative nausea/vomiting, total intravenous analgesic dose, length of hospitalization, and complications.

Vomiting was treated with intravenous metoclopramide 10 mg as needed. The need for analgesia in the early postoperative period was determined by vital signs (tachycardia and hypertension) and patient irritability; thereafter it was employed when the patient scaled his/her pain ≥ 5 on a 10 point numerical rating scale (NRS). Analgesia was achieved by meperidine 0.5 mg/kg intravenously as required. The total dose of analgesia was considered as an indicator of pain severity.

Criteria for discharge included full consciousness and stable vital signs in the absence of any discomfort, vomiting or bleeding.

Data were analysed using SPSS version 16.0. Mean values were compared using t-test and frequency values were compared using Chi-Square test. Multivariate analyses were performed for controlling confounding factors. Statistical significance was set at P < 0.05.

## RESULTS

The study included 30 patients consisting of 22 (73.3%) male and 8 (26.6%) female subjects with mean age of 49.4 ± 16.3 (range, 23–76) years. No significant difference was present between the two groups in terms of age or sex (Table 1) implying satisfactory randomization.

Eighteen patients had RD involving ≤ 3 clock hours while the other 12 subjects had RD extending more than 3 but less than 5 clock hours. The size of the retinal break in all patients was less than one clock hour and there was no sign of chronicity or PVR in any of the eyes. Intraocular pressure (IOP) was less than 18 mmHg in all patients without any sign of glaucomatous optic neuropathy.

The extent of RD and breaks were similar in the study groups; therefore the procedure and the amount of cryopexy were comparable in both groups. Corneal epithelial debridement was not performed in any eye during surgery.

Table 2 summarizes the main outcome measures. Rates of OCR and PONV were significantly lower and the severity of the OCR was significantly less in the lidocaine group. Mean total analgesic dose was also significantly less in the lidocaine group. However, there was no significant difference between the study groups in terms of duration of surgery and hospital stay. No postoperative complications such as IOP elevation, uveitis, secondary RD or non-attachment were noted.

## DISCUSSION

Based on the results of the current study, local adjunctive application of lidocaine under GA can reduce the rate of the OCR and decrease its severity in patients undergoing SB. Moreover, the application of lidocaine significantly decreased PONV which is one of the most common postoperative complications of retinal detachment surgery. Such therapy was also associated with a reduced dose of IV analgesics. However despite these advantages, the duration of surgery and mean hospital stay were not different from the control group.

To the best of our knowledge, no other study has evaluated the adjunctive use of local lidocaine on the incidence of intra- and postoperative complications of scleral buckling procedures. There is one study on the use of lidocaine during vitrectomy and strabismus surgery. Ruta et al^36^ evaluated the influence of locally administered lidocaine on the rate of the OCR in 140 patients undergoing strabismus or retinal surgery. All patients received standard premedication and anesthesia, and were randomly assigned to receive 1mg/kg lidocaine applied locally to the muscles after conjunctival opening versus the same volume of saline (0.9%). Surgical stimulation occurred 5 minutes after administration of the drug. This study showed that local application of lidocaine reduced the incidence of OCR (86.1% vs. 37.1%) and severe bradyarrhythmias (40% vs. 2.9%). Cardiac arrest longer than 10 seconds was not observed in the lidocaine group but occurred in 14.8% of cases in the control group. These results are similar to our findings; however the authors did not report any data on the duration of surgery, hospital stay, PONV or analgesic dose. Differences in the rates of OCR between their study and the present one could be related to difference in the nature of the procedures.

In another study, Snir et al^39^ evaluated the safety and efficacy of propofol sedation combined with subtenon anesthesia for strabismus surgery in 32 patients under general (n=16) or local (n=16) anesthesia. In the local anesthesia group, sedation was induced with a loading dose of midazolam, fentanyl and propofol followed by continuous infusion of propofol (3 to 6 mg/kg/hr) for deep sedation. Subtenon anesthesia was accomplished by injection of 3 to 4 ml of a 1/1 mixture of lidocaine 2% and bupivacaine 0.5%. General anesthesia was achieved after premedication with midazolam followed by fentanyl, esmeron-bromate, propofol and tracheal intubation. According to their results, the local anesthesia group had significantly shorter operative and anesthesia time, fewer episodes of OCR or arrhythmia/bradycardia requiring treatment, fewer early or late episodes of PONV, and less pain. This study showed the same results as ours in terms of OCR and PONV, however we observed no difference in terms of operation time.

Chhabra and colleagues^40^ studied the efficacy of subtenon block for providing perioperative analgesia in 200 children undergoing vitreoretinal surgery. All subjects received either a subtenon block or 2 microgram/kg of intravenous fentanyl after induction of anesthesia in addition to instillation of proparacaine 0.5% drops on the conjunctiva. The incidence of OCR was significantly higher in the fentanyl group (31.6%) as compared to the subtenon block group (5.1%), but the incidence of PONV was similar. This study showed that subtenon block provides more effective analgesia than intravenous fentanyl in pediatric vitreoretinal surgery.

Ruta et al^36^ investigated the effect of local application of lidocaine to the extraocular muscles on the incidence of the OCR during strabismus surgery in 56 healthy children. They compared 3 groups: group 1 (n = 16), stimulation of the reflex without lidocaine; group 2 (n = 10), stimulation of the reflex after local application of 1 mg/kg of lidocaine 2% to the medial part of the eye following induction of anesthesia; group 3 (n = 30), stimulation of the reflex without and 5 minutes after application of lidocaine. Locally administered lidocaine significantly attenuated the OCR (105 bpm in group 2 vs. 86 bpm in group 1 and 82 vs. 63 bpm in group 3). Severe bradyarrhythmias, in particular cardiac stand-still, were not observed when lidocaine had been applied. No systemic side effects of lidocaine were seen. These results are in line with our observations in terms of OCR but the authors did not report other outcomes such as duration of surgery, hospital stay or PONV.

Gupta and colleagues^37^ randomly allocated 45 children with strabismus surgery to receive a peribulbar block, local lidocaine 2% combined with general anesthesia, or general anesthesia alone. Their results showed that the incidence and severity of the OCR was significantly reduced in children who received a peribulbar block. The incidence of PONV was significantly reduced in patients receiving peribulbar block or local anesthesia combined with general anesthesia as compared to general anesthesia alone. This study showed that peribulbar block is more effective than topical lidocaine 2% combined with general anesthesia in blunting the OCR but equally effective for reducing PONV; both of these methods were superior to general anesthesia alone. The findings of this study differ from ours in that the addition of local lidocaine to general anesthesia did not reduce the OCR. This difference might be attributed to differences in the type of surgery and continuous irrigation with lidocaine in our study.

In summary, our study revealed that local application of lidocaine 2% followed by continuous irrigation with lidocaine 1% during scleral buckling surgery for RRD under general anesthesia can decrease the rates of OCR, PONV, and also reduce analgesic dose. However it may not affect the duration of surgery and period of hospitalization.

## Figures and Tables

**Table 1:** Demographics of the study groups

	Lidocaine n=15	Control n = 15	P-value
Age (yr)	47.2±15.2	45.8±16.1	0.818*
Male/Female	10(67%)/5(33%)	12(80%)/3(20%)	0.341**

* Independent t-test;

** Chi-Square test

**Table 2:** Comparison of outcome measures between the study groups

		Lidocaine n = 15	Control n = 15	P-value
Rate of OCR	0	11 (73.3%)	3 (20%)	0.004*
	1	4 (26.6%)	6 (40%)	
	2	0 (0%)	6 (40%)	

Severe OCR		1 (6.6%)	6 (40%)	0.040*
PONV		1 (6.6%)	6 (40%)	0.040*
Analgesic Dose (mg)		3.6 ± 1.9	13.3 ± 3.6	0.031***
Surgery Duration (min)		76.0 ± 7.3	77.6 ± 9.2	0.589**
Hospital Stay (hours)		18.0 ± 2.0	18.0 ± 1.6	0.856**

OCR, oculocardiac reflex; PONV, postoperative nausea/vomiting

* Chi-Square Test;

** Independent t-Test;

*** Mann-Whitney Test
